# The Effects of 12-Week Traditional Thai Exercise (Ruesi Dadton) on Glycemic Control and Inflammatory Markers in Prediabetes: A Randomized Controlled Trial

**DOI:** 10.3390/life13112166

**Published:** 2023-11-05

**Authors:** Pornchai Sawangwong, Sucharat Tungsukruthai, Preecha Nootim, Kusuma Sriyakul, Pratya Phetkate, Kammal Kumar Pawa, Parunkul Tungsukruthai

**Affiliations:** 1 Chulabhorn International College of Medicine, Thammasat University, Pathum Thani 12120, Thailand; pornchai093@gmail.com (P.S.); kusuma_cat@yahoo.com (K.S.); pratya@tu.ac.th (P.P.); kammalkumarpawa@gmail.com (K.K.P.); 2 Division of Health and Applied Sciences, Faculty of Science, Prince of Songkla University, Hat Yai 90110, Thailand; sucharat.t@psu.ac.th; 3 Department of Thai Traditional and Alternative Medicine, Ministry of Public Health, Nonthaburi 11000, Thailand; preecha.nootim@gmail.com

**Keywords:** Ruesi Dadton, exercise, prediabetes, glycemic level, CRP, IL-6

## Abstract

Hyperglycemia and inflammation are hallmarks of the prediabetes stage, which has the potential to develop into diabetes mellitus. In this stage, lifestyle changes and exercise are recommended and have been shown to be effective. However, there has been insufficient study investigating the impact of Ruesi Dadton (RD) exercise on prediabetes. Therefore, this study aimed to investigate the effect of RD exercise on biomarkers of glycemic level including fasting plasma glucose (FPG), the 2 h oral glucose tolerance test (OGTT), hemoglobin A1C (HbA1C), the biomarkers of inflammation C-reactive protein (CRP) and interleukin 6 (IL-6), and body mass index (BMI) on prediabetes during 12-week RD exercise. A total of 64 participants were randomly assigned into two groups, RD and control (CON), and were tested by measuring their glycemic levels to screen for prediabetes. The RD group was instructed to perform 10 postures of RD exercise in 60 min, three times a week. The CON group received standard lifestyle recommendations that were not pharmacologically managed. The results reveal that the RD group experienced a significant decrease in FPG, OGTT, HbA1C, and IL-6 (*p* < 0.01), and BMI and CRP (*p* < 0.05) compared to the CON group. In addition, the CON group had considerably higher glycemic levels, BMI and IL-6 levels (*p* < 0.01). Our study demonstrates that RD could decrease the biomarkers of glycemic level and inflammation during 12 weeks of RD exercise in prediabetes. These findings suggest that RD exercise is an effective approach for reducing systemic inflammation and controlling glycemic levels in prediabetic patients.

## 1. Introduction

Type 2 diabetes mellitus (T2DM) is a chronic disorder that is regarded as one of the major global health issues. The prevalence of T2DM is influenced by a complex interplay of genetic, metabolic, and environmental risk factors [1]. According to the International Diabetes Federation (IDF), 463 million people worldwide have diabetes, with that number predicted to rise to 783.2 million by 2045 [2]. Moreover, individuals between the ages of 40 and 59 are most affected by diabetes [3]. Thailand has the seventh largest number of diabetic patients in the Western Pacific. Furthermore, diabetes accounts for around 30,000 deaths of all deaths due to chronic non-communicable diseases in Thailand [4,5].

The primary pathophysiological processes involved in the development of T2DM are a reduction in insulin secretion by β-cells and/or a decline in insulin sensitivity (insulin resistance), which causes the body to have high blood sugar levels (hyperglycemia) [6]. Chronic hyperglycemia or prolonged high blood sugar levels is related to the destruction, deterioration, and failure of fundamental organs [7]. Furthermore, long-term exposure to hyperglycemia, which causes oxidative stress and inflammation, may alter gene expression regulation, resulting in lessened insulin production and the increased apoptosis of β-cells [8].

Diabetes mellitus may be facilitated by several risk factors, including obesity, smoking, and hypertension [9]. In addition to the aforementioned variables, inflammation has been suggested as a mechanism in the development of T2DM [10]. Earlier research has revealed that inflammation plays a crucial role in the pathophysiology of T2DM and the development of insulin resistance [11]. Even minor changes in glucose levels have been associated with inflammatory processes and T2DM consequences [12]. Furthermore, previous research has found that inflammation is also associated with other illnesses related to T2DM, such as heart failure, atherosclerosis, metabolic syndrome, cardiometabolic diseases, renal diseases, and various cancers [13].

C-reactive protein (CRP), one of the sensitive indicators of systemic inflammation, has been suggested to be increased in patients with T2DM. Men with CRP levels ≥ 2.91 mg/L were found to have a 2.7 times higher risk of developing diabetes [14]. In addition, CRP is an effective independent marker of cardiovascular disease and the consequences of acute coronary syndrome [15]. CRP is controlled by adipocyte-derived proinflammatory cytokines, including tumor necrosis factor α (TNF-α) and interleukin 6 (IL-6) [16]. Moreover, IL-6, a proinflammatory cytokine, has been shown to induce gluconeogenesis subsequent to hyperglycemia and peripheral insulin resistance [17]. Outstandingly, increased circulating levels of inflammatory markers such as CRP and IL-6 have been linked to insulin resistance and progression to T2DM [18,19,20]. Therefore, to treat the disease, it is necessary to minimize the abnormally raised levels of inflammatory cytokines in diabetic patients.

Before developing T2DM, the majority of people go through a prediabetic stage [21]. Impaired fasting glucose (IFG) and impaired glucose tolerance (IGT) are key characteristics of this prediabetic stage. The risk of developing diabetes has been found to be higher in prediabetic patients who are obese and physically inactive than in other patients. Moreover, without early management, prediabetics have a 70% chance of developing diabetes [22]. Furthermore, prediabetes increases the risk of cardiovascular disease, including stroke and peripheral vascular disease, leading to more severe consequences [23]. Regarding IL-6, previous research found that levels in prediabetics were noticeably higher than levels in healthy individuals [24]. Moreover, increased serum CRP levels have been linked to prediabetes, specifically decreased glucose tolerance [25]. Taken together, it is of the upmost importance to identify and treat people with prediabetes as soon as possible.

Regular physical exercise is a lifestyle modification that is essential in the early management of prediabetes because of its cost-effectiveness and the fact that it prevents pharmacological side effects [26]. Exercise has been found to improve the absorption of glucose by the skeletal muscle and able to decrease levels of inflammatory markers in the blood [27,28]. Prior studies suggest that exercise and/or physical activity has an anti-inflammatory impact, which may prevent or delay the development of T2DM [29,30]. A recent study, for example, has shown that combining aerobic and resistance exercise might reduce plasma levels of the proinflammatory marker IL-6 by up to 25%, potentially reducing the negative health impacts of diabetes-related inflammation [31]. In addition, Qigong, a traditional Chinese exercise classified as an oriental exercise, can decrease blood sugar and HbA1C levels in diabetic patients [32,33]. Moreover, in another study, Qigong was found to improve sleep disturbance and depressive symptoms and reduce the levels of interleukin-1β (IL-1β) and IL-6 [34]. Furthermore, not only can it control an individual’s glycemic levels, but Chinese exercise can also decrease inflammatory markers such as TNF-α and IL-6 and CRP levels [35].

In Thailand, Ruesi Dadton (RD) is a traditional Thai mind–body workout and one form of oriental exercise that combines slow movement with deep breathing and breath-holding for mental and physical well-being [36]. Previous research has indicated some favorable effects of RD exercise on health outcomes, such as improving and promoting physical function, improving cognitive ability, decreasing oxidative stress, and improving cardiopulmonary function. In addition, RD can reduce stress, which allows for an improvement in quality of life and safety for practitioners [36,37,38,39]. However, the majority of the oriental exercise methods studied in prediabetes or diabetes concern Qigong. There is insufficient evidence regarding the ability of RD exercise to affect glycemic levels and biomarkers of inflammation in prediabetic patients. We hypothesize that glycemic levels and biomarkers of inflammation will decrease after RD exercise. Therefore, the aim of this study is to compare the effects of RD exercise and standard lifestyle recommendations that are not pharmacologically managed on glycemic levels, including FPG, OGTT, and HbA1C, as well as inflammation biomarkers, including CRP and IL-6.

## 2. Materials and Methods

### 2.1. Study Design and Participants

The study was designed as a single-blind (assessor) randomized control clinical trial, with a two-group design, the Ruesi Dadton (RD) group and standard lifestyle recommendations that have not been pharmacologically managed and the control group (CON), at the Thai Traditional and Integrated Medicine Hospital, Department of Thai Traditional and Alternative Medicine, and lasted for 12 weeks. The sample size was calculated according to a previous study [40]. The minimum sample size was estimated as 29 patients per group. However, to account for a 10% drop-out rate, we recruited a total of 32 patients per group.

The inclusion criteria were individuals aged 18–70 years old, with a body mass index (BMI) ≥ 23–29.90 kg/m^2^ and individuals diagnosed with impaired glucose regulation (IGR), fasting plasma glucose (FPG) 100–125 mg/dL, and 2 h plasma glucose (OGTT) ≥ 140–199 mg/dL or (hemoglobin A1c (HbA1c) 5.7–6.4%). The exclusion criteria were those participating in diabetes education programs, the consumption of herbal medicine within 60 days or another alternative that affects glycemic levels; regular exercise within 2 years (≥30 min/day; ≥3 days/week); osteoarthritis of the knee, spinal problems, such as scoliosis; and self-reported medical conditions, such as atherosclerotic cardiovascular disease; chronic kidney disease; heart failure; atrial fibrillation; stroke; peripheral vascular disease; rheumatoid arthritis; cancer; osteoporosis; depression; asthma; chronic obstructive pulmonary disease; dementia; severe mental illness; epilepsy; hypothyroidism; and learning disability. Participants who met the conditions and inclusion criteria had their blood collected by a blinded assessor to determine their blood glucose levels and inflammation biomarkers. Then, all participants were randomly divided using a stratified sampling method related to the variables of gender, age, FPG, and BMI by a computer program.

**Figure 1:**
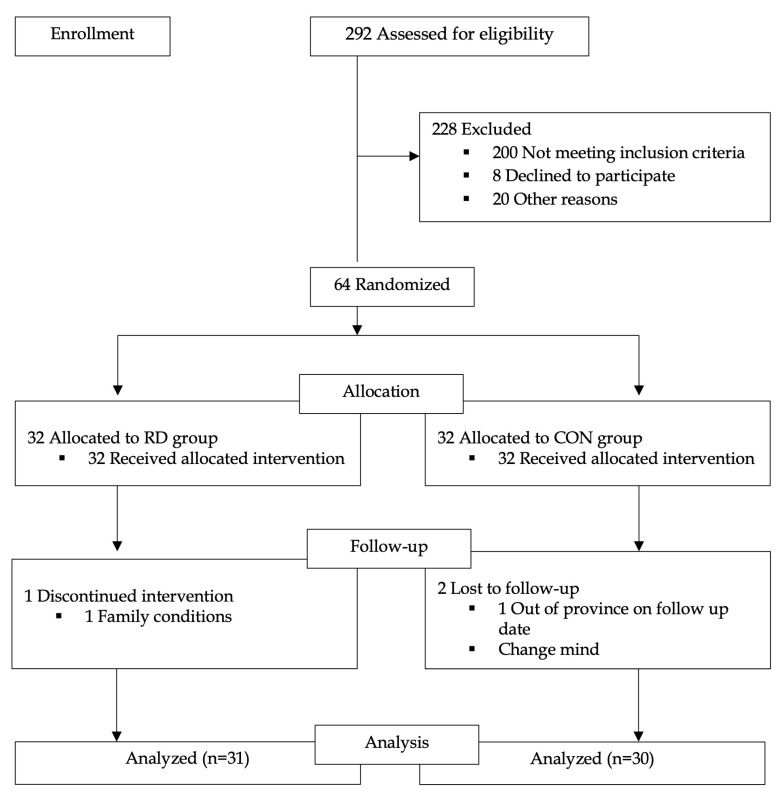
Study flowchart. Abbreviations: Ruesi Dadton (RD) and control (CON).

A total of 292 participants were initially screened (Figure 1). After excluding the samples that did not pass the criteria, glycemic and inflammation biomarkers were analyzed (RD group, *n* = 31, and CON group, *n* = 30). The experiment was carried out in accordance with the Declaration of Helsinki’s ethical guidelines. All participants were physically examined to confirm the absence of specific diseases before the experiment was conducted by a medical doctor and the patient provided written informed consent prior to participation. This study was approved by the Human Research Ethics Committee of Thammasat University (Medicine), Thammasat University, Thailand (approval no. MTU-EC-OO-0-036/64), and registered in the Thai Clinical Trials Registry (TCTR20230616004).

### 2.2. Exercise Intervention

The RD exercise protocol was performed as described in the literature [41]. Briefly, the RD exercise was demonstrated by a Thai traditional medicine (TTM) coach, during which the participants were taught and practiced breathing techniques for two days (60 min per day) on-site at the Thai Traditional and Integrated Hospital, Department of Thai Traditional and Alternative Medicine, Ministry of Public Health, Thailand. This was followed by a 12-week online intervention supervised by the TTM coach, which required the participants to perform 60 min of RD exercise, three times a week for 12 weeks totaling 36 sessions. The TTM coach was not blinded to the interventions due to the necessity of knowledge about the prescribed exercise for each RD exercise protocol. Likewise, it was not possible to blind the participants receiving the exercise interventions to the interventions. A video recording was sent to the TTM coach at the end of each session (movements were supervised and corrected by an online TTM coach). The main RD procedure included slow stretching, and combined with deep breathing, this involved holding one’s breath for about 10 s and then gently breathing out. An RD exercise program, with 10 RD postures used as an exercise intervention for prediabetes divided into three phases, needs to begin with a warm-up phase for 5 min (to relax and facilitate the joints and muscles of the body segments), after which the main exercise phase lasts for 50 min (to increase muscle strength and balance) and then a cool-down phase lasting 5 min takes place (to cool down and relax the muscles). The participants in both groups received information from the medical doctor about basic knowledge of prediabetes. The CON group were given a standard selfcare manual for prediabetes and could live their lives normally as before with no requirement to practice mind–body exercise until the intervention finished.

### 2.3. Blood Sample Analysis and Data Collection

All data, including FPG, OGTT, HbA1C, CRP, IL-6, and BMI, were recorded and collected by specialist staff according to the experimental operation manual and kept confidential to ensure the privacy of the participants. Then, the original data were entered, sorted, checked, and maintained by specialized data management personnel to ensure the accuracy and safety of the data. At the end of the 12th week, the research data were reviewed. Fasting venous blood samples following overnight fasting (~12 h other than water) were collected before and after 12 weeks of the RD exercise (10 mL) and used to determine biological markers of FPG, HbA1C, OGTT, CRP and IL-6. The blood samples were collected into a fluoride/ethylenediaminetetraacetate (EDTA) K2 plasma collection tube for the glycemic test and a plain (no additive) plasma collection tube for the inflammation biomarkers. OGTT, HbA1C, CRP, and IL-6 were measured pre- and post-intervention (12 weeks); meanwhile, FPG was measured every 4 weeks for 12 weeks. OGTT was measured following venous blood collection; the participants consumed a 75 g glucose-infused drink (UTOPIAN Co., Ltd., Samut Prakan, Thailand) for 120 min post consumption. CRP was tested by collecting serum samples by means of immunoturbidimetric testing with Beckman coulter CRP reagent using an automated machine. IL-6 was tested by collecting serum by means of ELISA and testing using an ELISA (R&D Systems, Inc., Minneapolis, MN, USA) reagent kit with an automated machine. The laboratory data were compiled at BANGKOK R.I.A. LAB Co., Ltd. (Bangkok, Thailand). For height and body weight, these factors were measured at baseline and after 12 weeks using a stadiometer and an electronic scale, both of which were calibrated annually. The measurements were taken twice by the same well-trained measurer using the auscultatory method. BMI was calculated at both baseline and post-intervention using the participants’ respective height and body weight with the following equation: BMI (kg/m^2^) = Body Weight (kg) ÷ Height (m)^2^.

### 2.4. Study Outcomes

The primary study outcome was glycemic levels, including FPG, OGTT, and HbA1C. The secondary outcomes were inflammation biomarkers, including CRP, IL-6, and changes in BMI. 

### 2.5. Statistical Analysis

This study analyzed the data produced by utilizing the per-protocol method. Every step of the statistical analyses was performed using SPSS version 24 (IBM Corp., Armonk, NY, USA). The characteristics of the participants were analyzed and reported as frequencies, percentages, and mean ± SD. Fisher’s exact test and independent *t*-tests were conducted to assess the differences between the two independent means. All outcome variables were tested for normality using the Shapiro–Wilk and Kolmogorov–Smirnov tests. Power analysis calculations were carried out for a paired sample *t*-test for the difference between two dependent means, and they were also carried out for an independent *t*-test for the difference between two independent means. Change values were calculated by subtracting baseline values from the week 4, week 8, and week 12 values using repeated measures analysis of variance (ANOVA), followed by post hoc comparison analysis. *p*-values less than 0.05 were considered statistically significant.

## 3. Results

### 3.1. Baseline Characteristics

The baseline demographic characteristics of the participants are presented in Table 1. Sex, age, BMI, FPG, OGTT, HbA1C, CRP, and IL-6 were not significantly different. The levels of FPG and OGTT in both groups were less than 125 mg/dL and 199 mg/dL, respectively. Furthermore, because OGTT is more sensitive than HbA1c in Thai people [21], we used OGTT as the main criteria in this study to demonstrate that all participants had prediabetes at baseline.

### 3.2. The Effect of RD Exercise on FPG Levels

Table 2 reports the results of the repeated measures ANOVA used to assess group differences in the dependent variables at different time points. In the RD group, the levels of FPG significantly decreased from 110.00 ± 6.89 at baseline to 91.10 ± 7.97 at week 12 (*p* < 0.01). In contrast, the levels of FPG in the CON group significant increased (*p* < 0.01) from 106.63 ± 6.34 to 109.20 ± 9.17 mg/dL at week 12. In addition, there were significant differences in the FPG levels between the groups at week 8 and 12. At week 8, the level of FPG in the RD group was 97.58 ± 9.34 mg/dL, whereas the level of FPG in the CON group was 104.70 ± 10.69 mg/dL (*p* < 0.01). In addition, according to week 12, the level of FPG in the RD group was 91.10 ± 7.97 mg/dL, whereas the level of FPG in the CON group was 109.20 ± 9.17 mg/dL (*p* < 0.01). Taken together, the results show that performing RD exercise can gradually decrease FPG levels in prediabetics.

### 3.3. The Effect of RD Exercise on OGTT and HbA1C Levels

As shown in Table 3, in both groups, the levels of OGTT and HbA1C were measured pre- and post-intervention. When compared to the CON group, the post-treatment data showed that the RD group had significantly reduced levels of OGTT and HbA1C (*p* < 0.01). In terms of OGTT, its value significantly decreased from 169.81 ± 18.69 mg/dL at pre-treatment to 136.10 ± 28.57 mg/dL at post-treatment (*p* < 0.01). However, in the CON group, the levels of OGTT significantly increased from 165.13 ± 23.36 mg/dL at pre-treatment to 180.00 ± 34.44 mg/dL at post-treatment (*p* < 0.01). In terms of HbA1C, RD exercise significantly decreased levels from 5.68 ± 0.33 at pre-treatment to 5.45 ± 0.38 at post-treatment (*p* < 0.01). However, in the CON group, the results showed that the level of HbA1C significantly increased from 5.69 ± 0.39 at baseline to 5.76 ± 0.42 at post-intervention (*p* < 0.01). As a result, RD exercise could improve glycemic levels to a greater extent.

### 3.4. The Effect of RD Exercise on Circulating Inflammatory Markers

Studies in the past have indicated that inflammation is fundamental to the pathophysiology of T2DM and the emergence of insulin resistance [42,43]. In particular, CRP and IL-6 have been used as significant indicators in the transition from the prediabetic state to the diabetic state [44]. As a result, the decrease in inflammatory markers could attenuate the progression of comorbid conditions in prediabetic patients.

In this study, the levels of inflammatory markers such as CRP and IL-6 were examined before and after treatment, as shown in Table 4. When compared to the CON group, the CRP levels in the RD group significantly decreased (*p* < 0.05). The CRP levels in the RD group at post-intervention were 1.68 ± 1.11 mg/L. On the contrary, the CRP levels in the CON group were 2.25 ± 0.99 mg/L. In addition, IL-6 levels were significantly reduced in the RD group compared to the CON group (*p* < 0.01). The IL-6 levels in the RD group at post-intervention were 1.74 ± 0.56 mg/L. On the other hand, the IL-6 levels in the CON group were 3.67 ± 2.39 mg/L.

Interestingly, the results from the CON group showed that the CRP and IL-6 levels, in particular CRP, increased from 2.09 ± 1.07 to 2.25 ± 0.99 and from 2.24 ± 0.71 to 3.67 ± 2.39, respectively. When considered collectively, RD exercise demonstrated significant protective effects against diabetes-related inflammation, suggesting that it might be used as an alternative method to promote physical health.

### 3.5. The Effect of RD Exercise on BMI

In this study, the BMI values of the patients were examined before and after treatment, as shown in Table 5. When compared to the CON group, the BMI values in the RD group significantly decreased (*p* < 0.01). The BMI values in the RD group at post-intervention were 25.51 ± 2.93 kg/m^2^. On the contrary, the BMI values in the CON group were 27.70 ± 2.78 kg/m^2^.

## 4. Discussion

Thailand has experienced an increase in the number of diabetic patients as a result of poor diets, high obesity rates, and an aging population. Diabetes significantly increases mortality and healthcare expenses and lowers quality of life, placing a heavy burden on developing nations [5]. Therefore, it was important to research alternative or interventional methods that could be used as early therapy for prediabetics or healthy individuals to delay and avoid the onset of T2DM. Regular physical exercise is one of the most effective treatments for preventing T2DM since it is cost-effective and prevents pharmaceutical side effects [1]. Ruesi Dadton (RD) is a traditional Thai mind–body workout that is commonly used by the elderly because it integrates slow movement with deep breathing. RD exercise has favorable effects on various body functions [38]. However, there is an absence of information examining the effects of RD exercise on preventing the progression of T2DM in prediabetics.

To the best of our knowledge, this is the first randomized controlled trial analyzing the impact of RD on glycemic levels and inflammatory profile in individuals with prediabetes. This study compared the benefits of RD exercise with conventional lifestyle suggestions that did not involve the use of medication on glycemic levels, such as FPG, OGTT, and HbA1C, as well as inflammation biomarkers, such as CRP and IL-6, in prediabetic patients. Our findings suggest that RD exercise significantly reduced the levels of CRP and IL-6 in prediabetic patients. In addition, the results of this study indicate that the RD exercise program had positive effects on reducing FPG, OGTT, and HbA1c levels in individuals with prediabetes.

According to several systematic reviews and meta-analyses, exercise could help to significantly enhance insulin sensitivity and improve glucose absorption in muscles and adipocytes in prediabetic and T2DM patients [45,46]. Furthermore, a recent study discovered that in patients with prediabetes, appropriate physical activity might decrease the risk of developing diabetes [47]. Wang et al. reported that participating in aerobic exercise could significantly lower glucose levels in individuals with prediabetes. Additionally, this might result in declines in BMI, FBG, 2hPG, HbA1c, and other relevant markers [48]. A previous study reported that Yijinjing, traditional Chinese exercise combined with resistance training, could improve fasting blood glucose, insulin resistance, and reduce liver fat in middle-aged and older people with prediabetes [49]. Moreover, earlier research revealed that Baduanjin Qigong, a traditional exercise that originates from ancient China, had significant effects on fasting blood glucose, HbA1c, and postprandial blood glucose. In addition, Qigong demonstrated better control of HbA1c than other forms of aerobic exercise [33,50].

Baduanjin Qigong is comparable to RD exercise in that it combines body movement, meditation guidance, and respiratory regulation [51]. In addition, RD exercise could decrease body weight, body mass index, and blood pressure while also enhancing quality of life [38]. According to our findings, prediabetic individuals who enrolled in the RD exercise program had lower FPG, OGTT, and HbA1c levels, whereas those in the CON group showed significantly increased glycemic levels (Table 2 and Table 3). For example, the OGTT levels in the RD group significantly decreased from 169.81 ± 18.69 to 136.10 ± 28.57 mg/dL (*p* < 0.01) whereas those in the CON group showed a significant increase from 165.13 ± 23.36 to 180.00 ± 34.44 (*p* < 0.01). These findings provide strong evidence that RD exercise could assist prediabetics in managing their glycemic levels. In light of the aforementioned results, the researchers would like to suggest that RD exercise should be highlighted as an alternative form of exercise for prediabetic patients.

Obesity and type 2 diabetes (T2DM) are linked by decreased exercise levels, decreased physical activity, and increased sedentary behaviors, which are linked to elevated indicators of persistent low-grade systemic inflammation [18]. Low-grade chronic inflammation has been proposed as the fundamental factor underlying T2DM, insulin resistance, and cardiovascular disease [42,43]. TNF-α and IL-6, which are generated by adipose tissue as adipokines, encourage low-grade systemic inflammation, which has been linked to chronic, detrimental conditions, such as T2DM, insulin resistance, and obesity [52]. Moreover, high CRP levels were found to be associated with a 2.2- and 8.1-times higher risk of developing prediabetes and T2DM, respectively, even after removing potential confounders, such as age, gender, and BMI [20,53]. By activating nuclear factor kappa B (NF-κB), a rise in CRP might cause apoptosis in β-cells and contribute to insulin resistance [54,55]. As a result, lowering the level of inflammation in the body associated with diabetes is crucial for preventing and regulating its progression. Our research demonstrates that RD exercise could dramatically lower the levels of CRP and IL-6 in prediabetic patients (Table 4), which could effectively lower the inflammation state of T2DM and the damage caused by CRP and IL-6 to β-cells. Surprisingly, CRP and IL-6 levels appear to be significantly increased in the absence of RD exercise (Table 4). CRP and IL-6 both experienced an increase in their percentage of mean change, reaching 7.66% and 63.84%, respectively.

To support our idea, meta-analyses have reported that exercise could reduce inflammatory cytokines (CRP, TNF-α, and IL-6) in T2DM patients [28,30]. Additionally, a recent study reported that physical activity with or without dietary or lifestyle modification reduced the levels of IL-6 in individuals with prediabetes [56]. For the mechanism of action, we hypothesized that lowering the inflammation markers CRP and IL-6 could decrease the apoptotic cell death of β-cells through the inhibition of the NF-κB pathway due to the fact that this pathway is mostly activated when the body experienced inflammation [57]. In addition, there is the persistent presence of hyperglycemia-activated NF-κB, which generated the expression of numerous cytokines, chemokines, and cell adhesion molecules. Moreover, NF-κB has been found to play a key role in the pathogenesis of the vascular complications of diabetes [58].

Physical activity has a positive impact on reducing glycemia, improving insulin resistance, and decreasing body weight [30,47,48]. In this study, the control group increased CRP and IL-6 levels, whereas the RD group significantly decreased these levels in 12 weeks. In parallel with our findings, the meta-analysis reported that exercise significantly decreased inflammatory cytokine CRP and IL-6 in patients with type 2 diabetes after a brief period of exercise (12–14 weeks) [30].

Previous study found that a higher BMI is associated with higher CRP concentrations [59]. As a result, we further analyzed the changes in BMI levels before and after the intervention, as shown in Table 5. BMI values in the RD group were observed to be considerably lower when compared to the control group (*p* < 0.01). Furthermore, a prior study discovered that in comparison to traditional exercise group, individuals in the control group (no exercise) had higher BMI values [60]. Moreover, when compared to lean patients, serum CRP levels in obese subjects increased significantly. Remarkably, CRP levels were associated with BMI and serum leptin levels [61]. Exercise appears to have a favorable effect on appetite via increasing peripheral and central leptin signaling (reuptake), particularly during weight loss [62].

**Figure 2:**
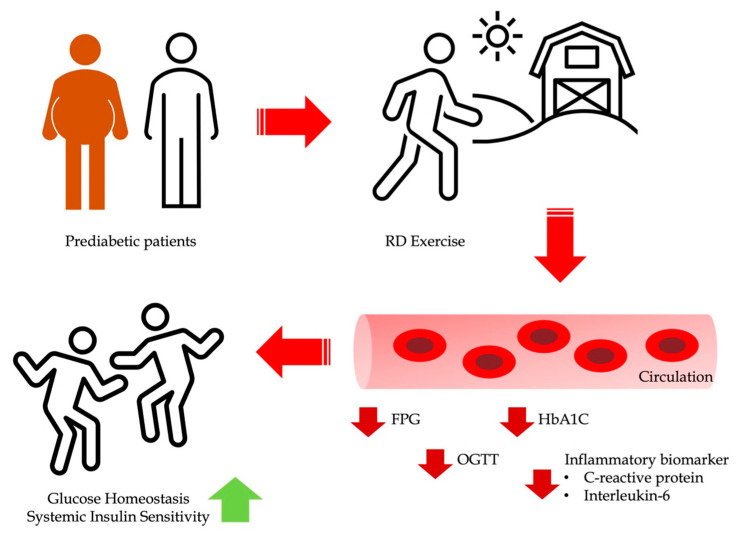
Scheme summary of the impact of RD exercise on glycemic levels and inflammation markers in prediabetic patients. Abbreviations: Ruesi Dadton (RD), hemoglobin A1c (HbA1c), 2 h plasma glucose (OGTT), and fasting plasma glucose (FPG).

To determine the reduction in BMI values, we required all participants in the RD group to send a video recording to the Thai Traditional Medicine coach at the end of each session. The participants in RD group exercised in a total of 36 sessions for the intervention, resulting in the reduction in BMI values, whereas the control group significantly increased BMI values. A prior research study found that many people ignored the major benefits to their physical health because they did not participate in regular physical activity in the absence of social and professional assistance [63]. We expected that the lack of engagement to exercise appeared to contribute to an increase in body weight, accompanied by elevated CRP levels and other inflammatory cytokine. Taken together, RD exercise could be a beneficial option for improving aberrations in blood inflammation levels as well as glycemic levels in patients with diabetes (Figure 2).

Due to the fact that this is the first randomized controlled trial on the effects of RD exercise in prediabetic patients, there were several limitations to this study. Therefore, further investigations are still necessary. In this study, we primarily examined IL-6 and CRP levels. However, it is essential to investigate other inflammatory factors, such as IL-10, TNF-α, and adipokines, which are implicated in prediabetes. Additionally, a comparative analysis of the effects of RD exercise combined with other interventions, such as resistant starch (RS) intervention, should be considered in future research [64]. Furthermore, this study only evaluated the effects of RD exercise over a 12-week period, and a more substantial representation of females and a study of longer duration should be carried out. Moreover, there is a further need for studies to assess the effects of RD exercise for T2DM patients.

## 5. Conclusions

In summary, the findings of this study revealed that RD exercise may be regarded as an effective training approach for assisting in the reduction in blood inflammatory factors, such as CRP and IL-6, while also lowering BMI and glycemic levels, including FPG, OGTT, and HbA1C, in the short term for prediabetic patients.

## Figures and Tables

**Table 1:** Baseline characteristics of the participants included in this study (mean ± SD).

Variables	RD (*n* = 31)	CON (*n* = 30)	*p*-Value
Sex ^a^			0.112
Male	23 (74.2%)	27 (90%)	
Female	8 (25.8%)	3 (10%)	
Age (years)	49.31 ± 13.01	51.16 ± 11.92	0.804
BMI (kg/m^2^)	26.06 ± 2.50	26.77 ± 2.72	0.218
FPG (mg/dL)	110.00 ± 6.89	106.63 ± 6.34	0.052
OGTT (mg/dL)	169.81 ± 18.69	165.13 ± 23.26	0.390
HbA1C (%)	5.68 ± 0.33	5.69 ± 0.39	0.892
CRP (mg/L)	2.38 ± 0.91	2.09 ± 1.07	0.260
IL-6 (pg/mL)	2.36 ± 0.77	2.24 ± 0.71	0.503

^a^ Fisher’s exact test.

**Table 2:** Change in FPG levels at different time points (mean ± SD).

Group	FPG (mg/dL)				*F*-Value	^a^ *p*-Value
	Baseline	Week-4	Week-8	Week-12		
RD (*n* = 31)	110.00 ± 6.89	98.45 ± 9.26	97.58 ± 9.34	91.10 ± 7.97	61.531	<0.001 **
CON (*n* = 30)	106.63 ± 6.34	99.73 ± 11.82	104.70 ± 10.69	109.20 ± 9.17	11.457	<0.001 **
^b^ *p*-value	0.052	0.638	0.007 **	<0.001 **		

** *p* < 0.01 ^a^ within group; ** *p* < 0.01 ^b^ between group.

**Table 3:** Change in OGTT and HbA1C levels pre- and post-intervention (mean ± SD).

Randomized Groups	RD (*n* = 31)	CON (*n* = 30)	*p*-Value
OGTT (mg/dL)			
Pre	169.81 ± 18.69	165.13 ± 23.36	0.390
Post	136.10 ± 28.57	180.00 ± 34.44	<0.001 ##
*p*-value %. mean change	0.002 ** ↓ 19.85%	0.001 ** ↑ 9.01%	
HbA1C (%)			
Pre	5.68 ± 0.33	5.69 ± 0.39	0.892
Post	5.45 ± 0.38	5.76 ± 0.42	0.004 ##
*p*-value %. mean change	<0.001 ** ↓ 4.05%	0.004 ** ↑ 0.07%	

** *p* < 0.01, compared with pre-intervention values within the group; ## *p* < 0.01, compared between the groups. The data were fitted into a normal distribution in each group.

**Table 4:** Change in inflammation levels pre- and post-intervention (mean ± SD).

Randomized Groups	RD (*n* = 31)	CON (*n* = 30)	*p*-Value
CRP (mg/L)			
Pre	2.38 ± 0.91	2.09 ± 1.07	0.260
Post	1.68 ± 1.11	2.25 ± 0.99	0.040 #
*p*-value % mean change	<0.002 ** ↓ 29.41%	0.504 ↑ 7.66%	
IL-6 (pg/mL)			
Pre	2.36 ± 0.77	2.24 ± 0.71	0.503
Post	1.74 ± 0.56	3.67 ± 2.39	<0.001 ##
*p*-value % mean change	<0.001 ** ↓ 26.27%	0.002 ** ↑ 63.84%	

** *p* < 0.01, compared with pre-intervention values within the group; # *p* < 0.05 and ## *p* < 0.01, compared between the groups. The data were fitted into a normal distribution in each group.

**Table 5:** Change in BMI values pre- and post-intervention (mean ± SD).

Randomized Groups	RD (*n* = 31)	CON (*n* = 30)	*p*-Value
BMI (kg/m^2^)			
Pre	26.06 ± 2.50	26.77 ± 2.72	0.218
Post	25.51 ± 2.93	27.70 ± 2.78	0.004 ##
*p*-value %. mean change	0.020 * ↓ 2.11%	<0.001 ** ↑ 3.47%	

* *p* < 0.05 and ** *p* < 0.01, compared with pre-intervention values within the group; ## *p* < 0.01, compared between the groups. The data were fitted into a normal distribution in each group.

## Data Availability

The data presented in this study are available within the article.
